# Corrosion Behavior in Volcanic Soils: In Search of Candidate Materials for Thermoelectric Devices

**DOI:** 10.3390/ma14247657

**Published:** 2021-12-12

**Authors:** Carlos Berlanga-Labari, Leyre Catalán, José F. Palacio, Gurutze Pérez, David Astrain

**Affiliations:** 1 Institute for Advanced Materials and Mathematics (INAMAT2), Engineering Department, Public University of Navarre, Campus Arrosadía, 31006 Pamplona, Spain; 2 Institute of Smart Cities (ISC), Engineering Department, Public University of Navarre, Campus Arrosadía, 31006 Pamplona, Spain; leyre.catalan@unavarra.es (L.C.); gurutze.perez@unavarra.es (G.P.); david.astrain@unavarra.es (D.A.); 3 Centre of Advanced Surface Engineering (AIN), Carretera de Pamplona 1, 31191 Cordovilla, Spain; jfpalacio@ain.es

**Keywords:** corrosion, soil corrosion, thermoelectricity, materials selection

## Abstract

Thermoelectric generators have emerged as an excellent solution for the energy supply of volcanic monitoring stations due to their compactness and continuous power generation. Nevertheless, in order to become a completely viable solution, it is necessary to ensure that their materials are able to resist in the acidic environment characteristic of volcanoes. Hence, the main objective of this work is to study the resistance to corrosion of six different metallic materials that are candidates for use in the heat exchangers. For this purpose, the metal probes have been buried for one year in the soil of the Teide volcano (Spain) and their corrosion behavior has been evaluated by using different techniques (OM, SEM, and XRD). The results have shown excessive corrosion damage to the copper, brass, and galvanized steel tubes. After evaluating the corrosion behavior and thermoelectric performance, AISI 304 and AISI 316 stainless steels are proposed for use as heat exchangers in thermoelectric devices in volcanic environments.

## 1. Introduction

At this exact moment, 10% of the global population is at risk of a volcanic eruption [1]. As most natural events, eruptions cannot be avoided. Nonetheless, volcanic vigilance has demonstrated to be able to predict when these eruptions are going to occur and, consequently, reduce their damage [2], becoming indispensable in any volcano in the world.

The biggest difficulty resides in supplying power to the necessary equipment, which constitutes a challenge since volcanic areas are usually remote, inaccessible, and lack power grid [3]. Normally, power supply is obtained by means of photovoltaic panels, transforming the solar radiation into electricity and storing it in a battery [4,5]. Nevertheless, this solution is not valid for all the volcanoes in the world. This is the case of those volcanoes that are located at high latitudes, and therefore, lack sun during months, or those that suffer of severe snowfalls that cover the PV panels, not permitting a continuous volcano vigilance [6,7].

One of the signs of volcanic activity is in the form of fumaroles, i.e., vents in the Earth’s surface from which steam and volcanic gases are emitted, typically at temperatures between 70 and 100 °C [8]. Hence, transforming this geothermal energy into electricity would permit obtaining a continuous power supply. For this purpose, since a low power supply is required compact, autonomous and robustly, binary cycles are not a viable option. As an alternative, thermoelectric generators have been proposed in the literature [9,10] due to their robustness and durability even without maintenance, as demonstrated in spatial applications [11]. Catalan et al. demonstrated their viability in reality, with a prototype that produced between 0.32 and 0.33 W in an 82 °C fumarole [12]. However, they realized that the conditions in which the generator needs to operate are extremely aggressive. In fact, as it is more deeply analyzed in Section 2, their device stopped its operation after only one week because of corrosion. Therefore, a critical aspect that must be taken into account for the durability of thermoelectric generators to become a completely valid solution is the use of materials that are resistant to corrosion in these harsh environments. 

Volcanic environments have aggressive chemistries due to the presence of gases like SO_2_(g) and H_2_S(g), and corrosive soils. Most of the existing studies focus on the effect of atmospheric corrosion in these environments, studying the corrosion products created and determining the most resistant materials for each type of volcanic environment [13,14,15,16,17,18]. In this work, instead of atmospheric corrosion, the effect of soil corrosion on different metallic materials has been analyzed in depth. Although the corrosion in soils is a phenomenon of great importance since it produces significant economic costs, few studies exist which analyses in detail the corrosion of buried metallic materials in soils. 

Galai et al. [19] carried out a comparative study of the corrosion resistance of copper alloys (copper, brass, and bronze) in a sandy soil in Essouari (Morocco). By carrying out tests on buried samples (160 days) and electrochemical tests during 21 days, they concluded that copper is the most resistant material to corrosion followed by bronze, while brass showed the worst behavior. On their behalf, Yan et al. [20] evaluated the corrosion resistance of a x80 steel pipe in an anoxic acid soil. Different moisture contents between 12% and 40% were used in electrochemical and gravimetric tests for 60 days. They highlighted that the maximum rate of corrosion (0.1 mm/year) was found for moisture contents of 30% or less.

Chung et al. [21] studied the influence of chloride, sulphate, and chloride concentration on the corrosion rate of a carbon steel pipeline in a natural soil by a statistical method according to the design of experiments (DOE). They concluded that the influence of the order of the independent variables on the corrosion rate was as follows: chloride concentration higher than sulphate concentration higher than pH. Moreover, a useful mathematical model was suggested.

Mahlobo et al. [22] studied the effect of cathodic protection polarisation on a carbon steel in an unsaturated soil. They observed that at −1.0 V vs. Cu/CuSO_4_, the effectiveness of the protection was a high measuring (7 µm yr^−1^); however, at 1.2 V Cu/CuSO_4_, the protective magnetite layer deteriorated after 63 days due to the formation of hydrogen bubbles. 

Nevertheless, until the moment, only Yurata et al. [23] have studied the resistance to the corrosion in volcanic soils. They evaluated the corrosion resistance of eight metallic materials in volcanic soils located on White Island in New Zealand at 40 and 100 °C for 111 days. The authors measured a high corrosion rate of more than 1 mm/year in the 400 steel, while the low alloy steels showed moderate resistance (0.1 mm/year). The stainless steels AISI 304 and 316 did not suffer and did not show any sign of corrosion. In line with this study, the present paper continues with the analysis of the resistance to corrosion of different materials, but considering a different environment: Teide volcano (Canary Islands, Spain), which presents a different soil composition as detailed in Section 2. The studied materials have also been different, specifically intended for thermoelectric generators. Furthermore, in order to have a better perspective of the durability of the materials, their exposure has been more prolonged: one year instead of 111 days. The objective of the present paper is to study the degradation of different materials buried in a volcanic soil, trying to determine which ones are more suitable for the heat exchangers of thermoelectric generators destined to autonomous volcanic vigilance stations. For this purpose, six different materials have been buried during one year at Teide volcano (Canary Islands, Tenerife, Spain), and several tests have been performed afterwards.

## 2. Materials and Methods

### 2.1. First Experience of Thermoelectric Generation in Fumaroles

Before delving into the degradation of different materials buried in volcanic soil, this section describes the first experience of thermoelectric generators in fumaroles, so that the present study is justified.

Thermoelectric generators are devices based on solid-state physics whereby heat is directly transformed into electricity thanks to Seebeck effect. This transformation is held in the so-called thermoelectric modules, which are made up of semiconductor materials united by a conductor material and isolated with a ceramic plate that also provides firmness to the modules. Since the efficiency of these modules is proportional to the temperature difference between their sides, it is necessary to incorporate heat exchangers with thermal resistances as low as possible so that the hot side of the modules approaches to the heat source temperature, and the cold side one to the ambient temperature.

Catalan et al. demonstrated that for geothermal shallow anomalies, the most suitable heat exchangers are those based on phase change [24]. This type of heat exchangers permit absorbing big amounts of heat and transport it almost isothermally, leading to low thermal resistances [25].

**Figure 1:**
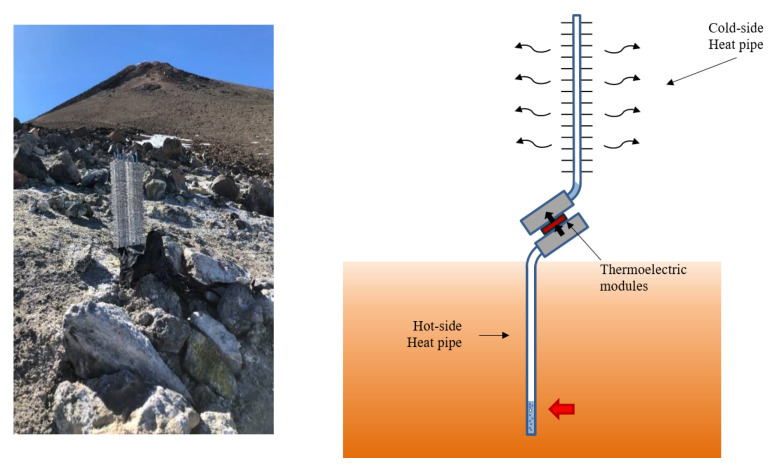
First thermoelectric generator installed at Teide volcano on March 2019 and its schema of operation.

Figure 1 depicts the first prototype of thermoelectric generator installed at Teide volcano in March 2019 [12], as well as its schema of operation. This device absorbs geothermal heat thanks to eight 450 mm long grooved heat pipes made of nickel-plated copper and with water as working fluid, which are inserted in an aluminum plate. Two bismuth-telluride thermoelectric modules transform part of this heat into electricity, releasing the rest to the environment by means of a similar heat exchanger, with the only difference of including 62 aluminum fins separated 5 mm for increasing the heat exchange area. 

**Figure 2:**
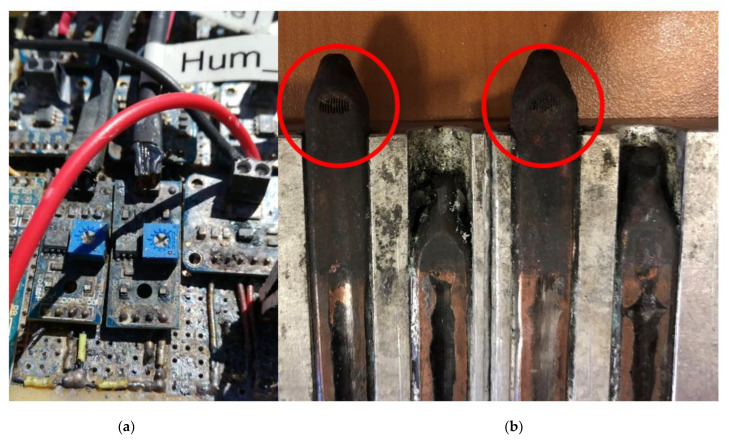
(**a**) Detail of the corrosion of the electronic system. (**b**) Detail of the perforated tubes due to corrosion.

This prototype was installed at Teide volcano (Canary Islands, Tenerife, Spain) on March 2019, at an altitude of 3500 m, where there exist 82.5 °C fumaroles. During three days, it demonstrated the viability of thermoelectric generation from fumaroles, being able to produce between 0.32 and 0.33 W, which represents a good starting point for autonomous volcanic monitoring. However, after just three days, the monitoring of the different variables stopped. The examination of the prototype three weeks later reported severe corrosion of the electronic system, as depicted in Figure 2. 

Due to the failure, the complete prototype was taken off, and thereafter, a visual analysis of corrosion in the generator itself could be performed. This analysis showed that corrosion also affected the generator. Copper tubes were the most affected element of the generator. In fact, as depicted in Figure 2, in only one month, corrosion perforated the tubes, causing the leakage of the internal working fluid.

Therefore, in order to be able to obtain robust and reliable thermoelectric generators for the stand-alone power supply of volcanic vigilance stations, it is necessary to make a deep study of which materials are able to withstand this acidic environment characteristic of volcanoes. This is indeed the objective of the present paper. 

### 2.2. Materials Tested and Soil Characteristics

**Figure 3:**
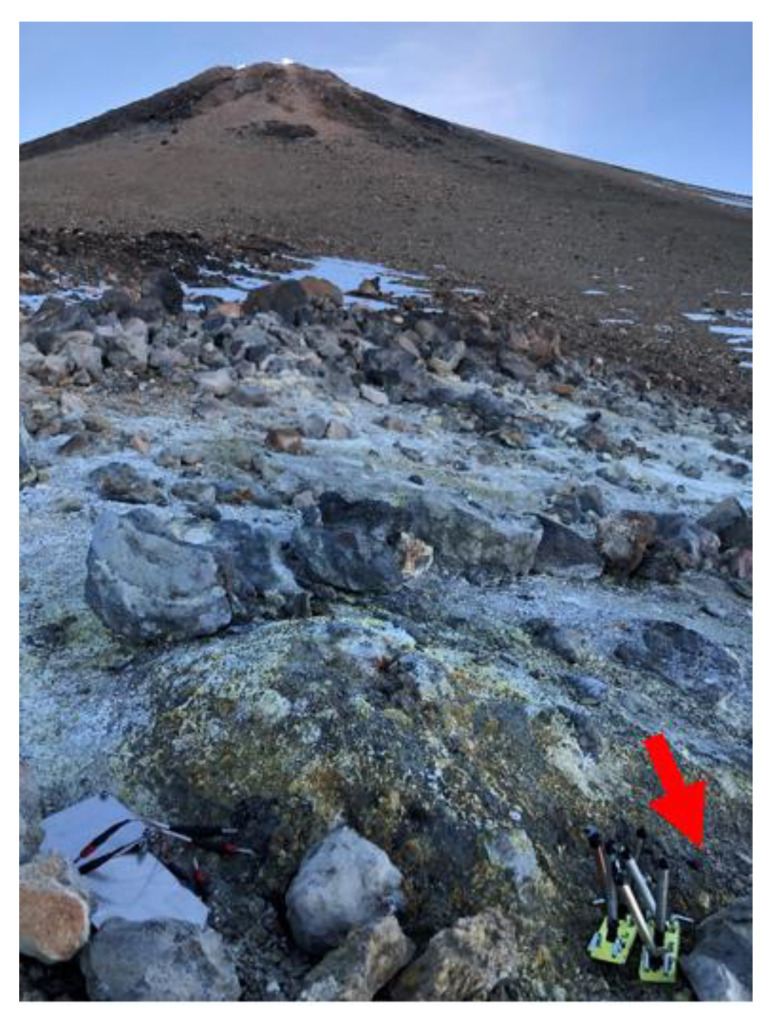
Location of the buried samples in the proximity a fumarole at Teide volcano.

In order to analyze which materials are more suitable for thermoelectric generators, different samples of six metals were buried during 12 months at Teide volcano at an altitude of approximately 3500 m, next to an 82.5 °C fumarole (Figure 3). More specifically, galvanized steel (thickness: 25 µm), anodized aluminum (thickness: 20 µm, anodized only in its interior), copper, brass (Zn: 36%), and stainless steels AISI 304 (Cr:18%, Ni:8%) and AISI 316 (Cr:18%, Ni:8%, Mo:3%) were selected. In all cases, 10 cm long tubes with a diameter of 12 mm were buried, since this is the predominant shape in thermoelectric generators with biphasic heat exchangers. The thickness of the probes varied between 0.5 and 1.5 mm due to the commercial availability. 

The soil in this area was formed from material of volcanic origin, made up of lava flows or products of aerial projection such as pyroclasts and ashes. This type of soil is mainly constituted by amorphous aluminosilicates and a high content of native sulphur. 

### 2.3. Methodology for Evaluation of Corrosion Damage

After the 12-month exposure to the volcanic environment, the samples were cleaned with different chemicals, in accordance with standard 8407 [26], to remove the soil and corrosion products adhered. Before and after the cleaning, the tubes were weighted using a precision balance in order to determine their weight loss. Afterwards, three cuts were performed and each of them was subject of a different analysis: metallographic analysis with an optical microscopy, X-ray diffraction, and surface morphology by SEM-EDX.

Optical microscopy observations (in bright field and under polarized light) were carried out using an Olympus metallographic microscope BX 60 (Tokyo, Japan). Before performing this analysis, the cross-section of each piece was prepared by grinding with silicon carbide abrasive papers ranging from 400 up to 2000 grit and polishing, with diamond pastes and alcohol-based lubricant, until a mirror-like surface was achieved. Then, the polished surface was cleaned in ethanol in an ultrasonic bath for few minutes to remove the residual abrasive particles.

For the X-Ray diffraction analysis, the crust formed on the external surface of the tubes was scrapped off with the help of a stainless steel spatula until sufficient amount of debris was collected. The debris was then smashed using an agate mortar to obtain a fine powder, and then placed on the XRD holder for the analysis. A Bruker D8 Discover equipment under Bragg Brentano configuration, using a Chromium source (Ka = 2.29 Å), was used (Billerica, MA, USA, 2012). For the identification of the peaks, the Crystallographic Open Database together with the data obtained from the EDX was of help.

The surface morphology was observed by means of a Hitachi S4800 scanning electron microscope (SEM) (Tokyo, Japan, 2006), which was coupled to an Inca EDX analyser (Oxford, United Kingdom, 2006) for chemical identification of the elements on the different surfaces. The acceleration voltage was 20 kV for all images and analysis, and both secondary electron and backscattered images were collected in order to obtain all the possible information from the surfaces.

## 3. Results

Once the methodology has been described, this section deals with the description of the results and their discussion. Firstly, a visual analysis is performed. This first analysis is followed by others that permit a more in-depth determination of the degradation: weight loss, metallography by optical microscopy, corrosion products by SEM-EDX, and X-ray diffraction. 

### 3.1. Visual Analysis of the Exposed Tubes

**Figure 4:**
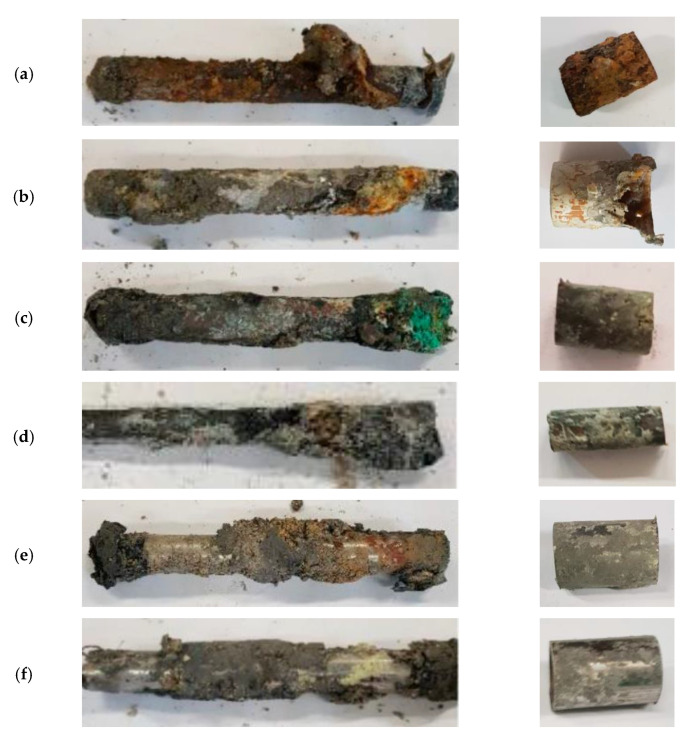
Photographs of the complete tubes after the field tests and a cut that was made for the subsequent analyses. (**a**) Galvanized Steel. (**b**) Anodized Aluminum. (**c**) Copper. (**d**) Brass. (**e**) AISI 304 and (**f**) AISI 316.

Figure 4 shows the six metal pipes after having been buried in the volcanic soil for one year, depicting the whole tube on the left and one cut that was made for the subsequent analyses on the right, before the chemical cleaning performed in accordance with standard 8407 [26].

As can be observed, all the samples show a high content of soil and corrosion products adhered to the interior and exterior of the tubes. When analyzed separately, the corrosion products present a different aspect. Thus, on the surface of the galvanized steel, a mixture of dark brown earths and white zinc corrosion products can be observed. In the anodized aluminum tube, a great amount of white corrosion products are detected. In addition, it is necessary to emphasize that in a zone of the tube, the corrosion has advanced from the interior achieving the tube fracture. Green and dark corrosion products mixed with earth have adhered to the copper tube. Brass shows less corrosion products and soil adhered to its surface. Finally, in the stainless steels 304 and 316 metallic zones without apparent corrosive damage can be observed.

Based on the visual analysis, the stainless steels are apparently the least corroded material. Nonetheless, it is necessary to perform more analyses in order to determine the degradation grade of each material, which is performed in the following subsections. 

### 3.2. Weight Loss and Microstructural Analysis of Exposed Samples

**Figure 5:**
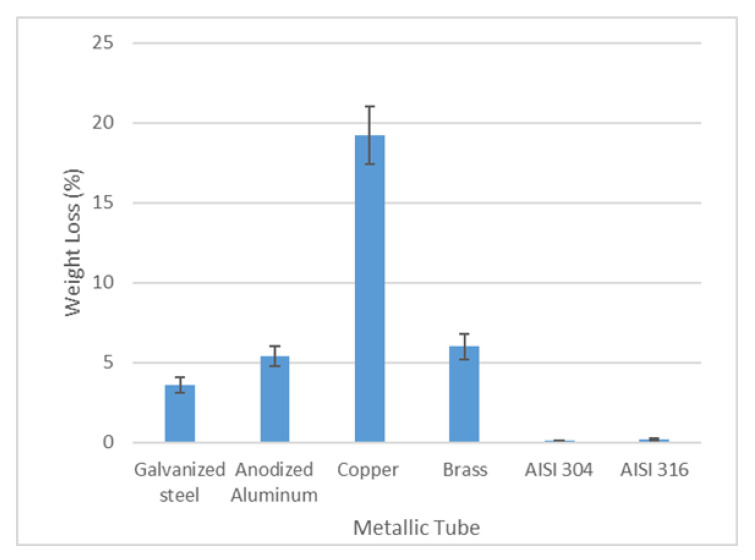
Representation of the weight loss of the metallic samples.

The initial analysis that was performed was the determination of weight loss, which was carried out with the whole samples (not cut). The results of weight variation after the field test are shown in Figure 5. As can be seen, copper is, by far, the material that has suffered the highest weight loss (19.25%) followed by brass (6%). The anodized aluminum and the galvanized steel have also undergone an important weight loss of 5.4% and 3.6%, respectively, while the stainless steels have not experienced any weight loss.

### 3.3. Corrosion Damage Evaluation by Optical Microscopy

**Figure 6:**
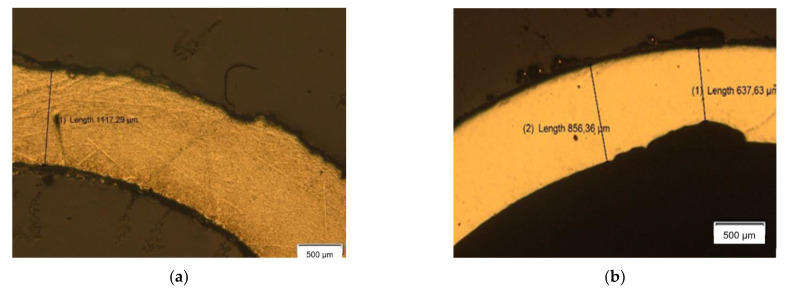

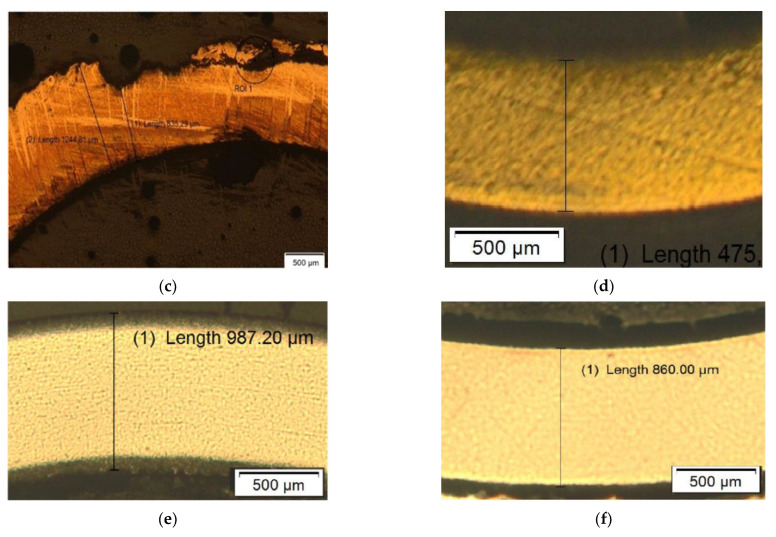
(**a**) Galvanized Steel. (**b**) Anodized Aluminum. (**c**) Copper. (**d**) Brass. (**e**) Stainless Steel AISI 304. (**f**) Stainless Steel AISI 316.

After the weight loss analysis, the samples were cut into smaller pieces in order to perform further studies. The first one consisted in a metallographic analysis with an optical microscopy, whose results are depicted in Figure 6.

These metallographies, in conjunction with Table 1, provide more information about the internal state of the exposed materials. Thus, in the galvanized steel sample corrosion damage of 0.3 mm can be found in some areas, which means a loss of thickness of more than 20%. Anodized aluminum also presents a high corrosive attack. Nonetheless, it occurs in the internal part, while the external surface has remained unaltered. This unique behavior is due to the fact that Teide’s volcanic soil presents a high content of native sulphur, which reacts with meteoric water leading to an acid soil. According to Pourboix diagrams, the corrosion resistance of aluminum decreases with a pH lower than 4, explaining its high corrosion rate in the interior of the tube, which was not anodized. The outer part of the sample presents Al_2_O_3_, leading to an elevated corrosion resistance in acidic environments. The rest of the samples present a more intense attack in their external surface. In fact, the entire external surface of the copper sample shows intense corrosion damage, in some cases resulting in a loss of thickness of 40%. On the contrary, the brass sample only shows some damage in localized areas of the exterior. Finally, the stainless steels 304 and 316 do not show signs of general or localized corrosion. 

### 3.4. Characterization Analysis of Corrosion Products

The next test that was performed to the samples was the analysis of the corrosion products by SEM-EDX.

The microanalyses carried out on the galvanized steel sample, as shown in Table 2, indicate the formation of mainly iron oxides. Titanium, silicon, sulphur and aluminum from the volcanic soil have also been detected, but in a lesser extent.

**Figure 7:**
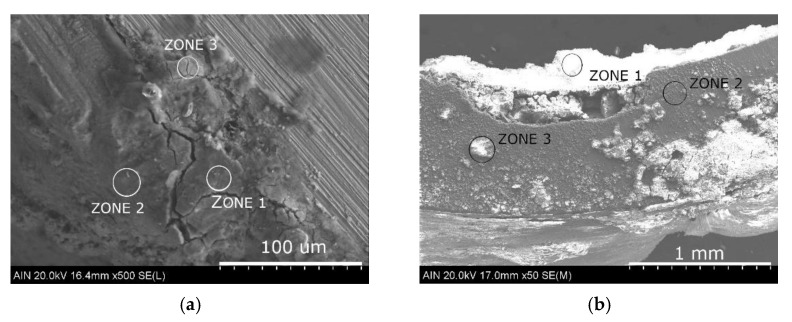
SEM images (**a**) in galvanized steel sample, (**b**) in anodized aluminum sample.

In the case of aluminum, the results vary depending on the zone of study as depicted in Table 2 and Figure 7. Thus, on the inner side of the aluminum sample (zone 1), a layer of silicates and sulphides from the soil can be seen. In zone 2, it can be seen how the corrosive attack progresses through the reaction of the soil components with the aluminum. In contrast, the anodizing layer of the aluminum sample has not suffered any corrosive damage and good adhesion to the aluminum is observed.

**Figure 8:**
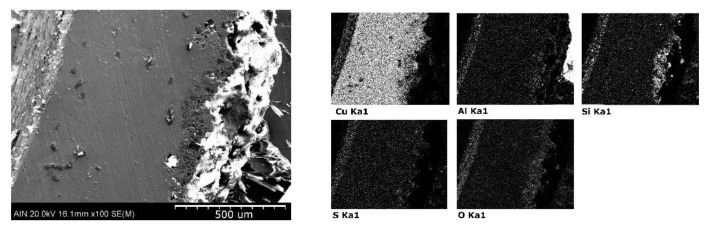
Elemental mapping: microanalysis by SEM-EDX performed of copper sample.

Figure 8 shows how the copper sample has undergone intense corrosive damage to the entire outer surface. More specifically, the results indicate that the copper reacts with the soil to form aluminum silicates and copper oxides.

**Figure 9:**
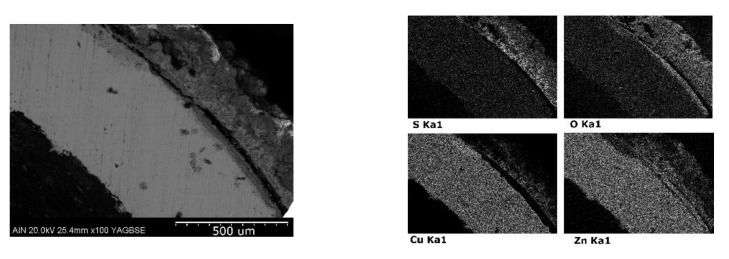
Elemental mapping performed by SEM-EDX in brass sample.

Figure 9 shows the formation of a large amount of oxides formed on the external surface of the brass due to the presence of moisture. The microanalyses carried out indicate that in the intercalary between the metal and the layer of oxides an impoverishment of the zinc has taken place, causing, therefore, a dezincification.

**Figure 10:**
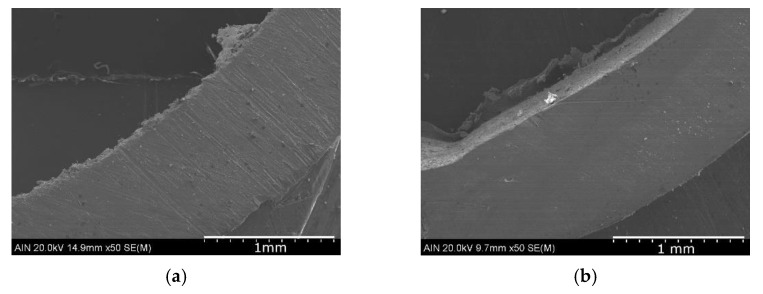
SEM images of (**a**) AISI 304 stainless steel, (**b**) AISI 316 stainless steel.

Finally, Figure 10 shows how a dense and adherent layer has formed on the inner surface of the AISI 304 and AISI 316 stainless steel samples. The external part has not experienced corrosion while the internal one has formed a layer of oxides, which is more intense in AISI 316.

**Figure 11:**
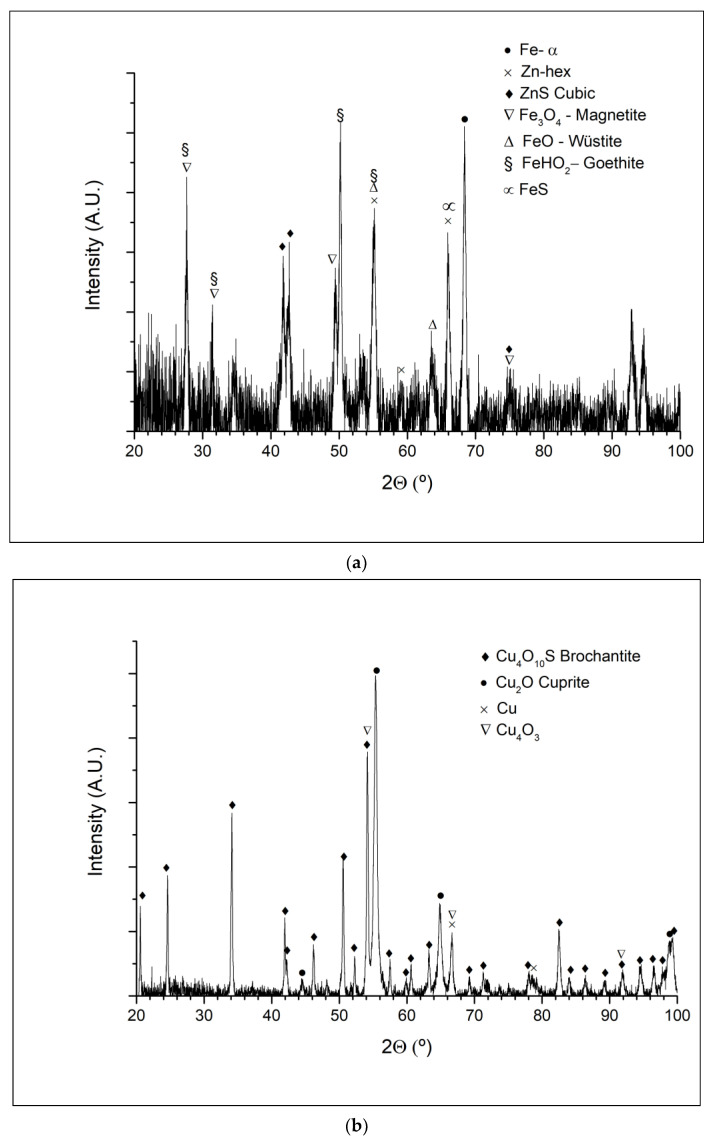

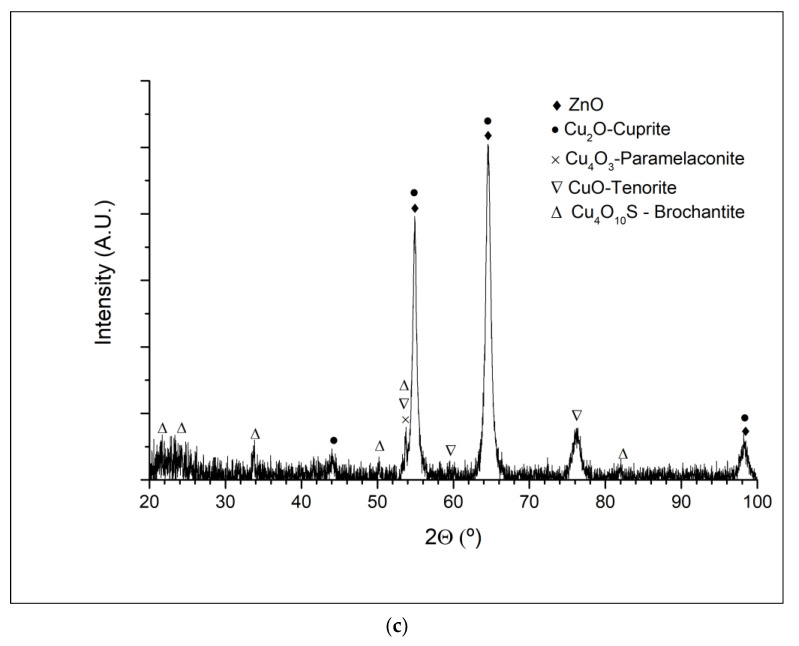
XRD difractogram of (**a**) galvanized steel, (**b**) copper, (**c**) and brass.

X-ray diffraction is the last analysis that was performed. Figure 11 displays the diffractograms obtained from the corrosion products removed from the galvanized steel, copper, and brass samples. Diffractograms of anodized aluminum and stainless steels were not obtained since insufficient corrosion products had been formed.

In the diffractogram of the galvanized steel a mixture of zinc corrosion products consequence of zinc and iron corrosion have been identified as FeO, Fe_3_O_4_, ZnS, and FeS_2_. 

On its behalf, the corrosion products identified in the copper sample have been mainly the cuprite (Cu_2_O) and paramelacomite (Cu_4_O_3_) and to a minor degree the brochantite (Cu_4_O_10_S).

Finally, in the corrosion products of brass, it has been mainly detected ZnO and to a lower proportion cuprite and tenorite.

### 3.5. Discussion

Based on the previous tests, this section jointly analyzes the obtained results in order to determine the most adequate material for thermoelectric generators.

The copper sample has suffered a severe corrosive attack resulting in an excessive loss of weight and thickness. The high content in sulphur compounds and low pH have led to the formation of abundant corrosion products such as Cu_2_O and CuS. The high corrosivity of this type of volcanic soils is mainly associated with the reactions of the native sulphur with the soil moisture producing hydrolysis reactions that lower the pH. Consequently, for future thermoelectric applications in volcanic environments, only copper protected with some type of organic coatings should be used. Otherwise, its use is not recommended.

The brass sample has suffered a selective corrosive attack due to the reaction of the zinc with the sulphur-rich compounds in the soil forming mainly ZnS. This dezincification corrosion mechanism, characteristic of brass alloys with zinc contents higher than 30%, was observed by the Greeks on brass buried in Roman times in the 10th century.

Similarly, galvanized steel has also experienced an excessive corrosive attack by reaction of zinc with sulphur compounds in the soil, forming again mainly ZnS.

The advance of corrosion on the inner side of the aluminum pipe has been very intense and has even perforated the pipe completely. According to the Pourbaix diagram of aluminum, at pH less than 4, aluminum behaves actively and therefore the protective layer of Al_2_O_3_ does not form. In contrast, the external anodized coating has performed well since its integrity has not been preserved and no corrosion products have formed.

Due to the formation of a passive layer, stainless steel samples have shown good corrosion performance in this acidic ambient so they are proposed as candidates for thermoelectric devices. 

## 4. Effect on Thermoelectric Generation

According to the results of the previous analyses, it becomes evident that copper cannot be again used as material in the heat exchangers of the thermoelectric generator. The best candidates are stainless steels A304 and A3016L. These materials are more resistant to the volcanic environment. However, their thermal conductivity is lower, which can diminish the generation of the thermoelectric generators. Therefore, in this section, the effect of using stainless steel tubes is briefly studied thanks to the computational model developed by Catalan et al. in [27] which presents errors lower than 8% in the estimation of power generation. 

**Figure 12:**
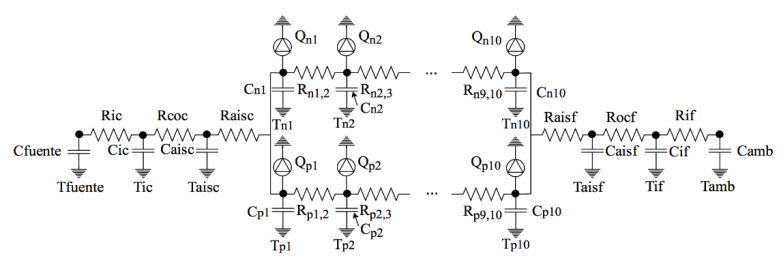
Thermal-electrical analogy for the computational simulation of a geothermal thermoelectric generator, with the hot heat exchanger on the left and the cold side one on the right.

Figure 12 shows the thermal-electrical analogy used for the former estimation. Using stainless steel instead of copper affects the conduction thermal resistances, increasing their value 25 times:

$$R_{k}=\frac{\ln\left(De/Di\right)}{2\cdot \pi \cdot L\cdot k}$$

Hence, reducing the thermal conductivity of the tubes from 385 to 14.4 W/m·K supposes a reduction in the power generation of 0.38 W, at 7.3%. Therefore, since this reduction is considerably low, the gain in robustness is worth it. 

## 5. Conclusions

In the present paper, the performance in operation of six candidate materials for use as heat exchangers in thermoelectric generators have been studied in a long-term test (one year) in the volcanic environment of Teide (Tenerife, Spain).

The copper, brass, and galvanized steel tubes have suffered a severe corrosive attack due to the physicochemical conditions of the volcanic soil. Thus, for their use as heat exchangers in future thermoelectric devices, these materials must be protected with an organic coating to achieve proper operation.

In contrast, the stainless steels AISI 304 and AISI 316L have shown a remarkable performance in service without suffering corrosion damage. In spite of having a lower thermal conductivity than the rest of the alloys, the power loss is only 7% compared to the calculations carried out with copper. Therefore, although the cost of these stainless steels is higher than the rest of alloys, their use is recommended as materials for the heat exchangers of the thermoelectric devices. 

To complete the study on the corrosion resistance of the materials of the thermoelectric devices, it would be necessary to evaluate the atmospheric resistance of the materials in contact with the fumaroles present in volcanic environments.

## Figures and Tables

**Table 1:** Values of the initial thickness and the average thickness after exposure of the different samples.

Sample	Initial Average Thickness (mm)	Average Thickness after Exposure (µm)	Standard Deviation after Exposure (µm)
Galvanized Steel	1.28	1228.84	71.51
Anodized Aluminum	0.86	809.59	62.59
Copper	1.42	1002.92	197.59
Brass	0.52	478.83	13.06
Stainless Steel A304	0.99	989.03	14.64
Stainless Steel A316L	0.86	857.71	7.59

**Table 2:** Values of elemental microanalysis corresponding to zones showed in Figure 7.

**Galvanized steel**	**Fe**	**Ti**	**F**	**Al**	**Si**	**S**	**K**	**O**	**Ca**
Zone 1	50.4		18.8		3.1	2.6		25.0	
Zone 2	57.2			19.1				23.6	
Zone 3	64.7					2.9		32.4	
**Anodized aluminum**									
Zone 1	4.8	3.6	0.4	40.6	41.8	7.8	1.1		0.4
Zone 2	8.2			67.3				24.3	
Zone 3	6.3	11.3			23.8	11.8			42.8

## Data Availability

Data sharing is not applicable to this article.
